# Potential of Host Markers Produced by Infection Phase-Dependent Antigen-Stimulated Cells for the Diagnosis of Tuberculosis in a Highly Endemic Area

**DOI:** 10.1371/journal.pone.0038501

**Published:** 2012-06-05

**Authors:** Novel N. Chegou, Paulin N. Essone, Andre G. Loxton, Kim Stanley, Gillian F. Black, Gian D. van der Spuy, Paul D. van Helden, Kees L. Franken, Shreemanta K. Parida, Michel R. Klein, Stefan H. E. Kaufmann, Tom H. M. Ottenhoff, Gerhard Walzl

**Affiliations:** 1 DST/NRF Centre of Excellence for Biomedical Tuberculosis Research and MRC Centre for Molecular and Cellular Biology, Division of Molecular Biology and Human Genetics, Department of Biomedical Sciences, Faculty of Health Sciences, Stellenbosch University, Tygerberg, South Africa; 2 Department of Infectious Diseases, Leiden University Medical Centre, Leiden, The Netherlands; 3 Department of Immunology, Max Planck Institute for Infection Biology, Berlin, Germany; McGill University, Canada

## Abstract

**Background:**

Recent interferon gamma (IFN-γ)-based studies have identified novel *Mycobacterium tuberculosis (M.tb)* infection phase-dependent antigens as diagnostic candidates. In this study, the levels of 11 host markers other than IFN-γ, were evaluated in whole blood culture supernatants after stimulation with *M.tb* infection phase-dependent antigens, for the diagnosis of TB disease.

**Methodology and Principal Findings:**

Five *M.tb* infection phase-dependent antigens, comprising of three DosR-regulon-encoded proteins (Rv2032, Rv0081, Rv1737c), and two resucitation promoting factors (Rv0867c and Rv2389c), were evaluated in a case-control study with 15 pulmonary TB patients and 15 household contacts that were recruited from a high TB incidence setting in Cape Town, South Africa. After a 7-day whole blood culture, supernatants were harvested and the levels of the host markers evaluated using the Luminex platform. Multiple antigen-specific host markers were identified with promising diagnostic potential. Rv0081-specific levels of IL-12(p40), IP-10, IL-10 and TNF-α were the most promising diagnostic candidates, each ascertaining TB disease with an accuracy of 100%, 95% confidence interval for the area under the receiver operating characteristics plots, (1.0 to 1.0).

**Conclusions:**

Multiple cytokines other than IFN-γ in whole blood culture supernatants after stimulation with M.tb infection phase-dependent antigens show promise as diagnostic markers for active TB. These preliminary findings should be verified in well-designed diagnostic studies employing short-term culture assays.

## Introduction

The diagnosis of tuberculosis (TB) remains a challenge in resource-constrained settings. In the absence of culture facilities, laboratory diagnosis of the disease is often only possible by Ziehl Neelsen-stained sputum smears, a test whose limitations are well known [1]. The introduction of the recently developed automated real-time sputum processing molecular beacon assay, the XpertMTB/RIF assay (Cepheid Inc., CA, USA) into clinical practice, is a significant development as the test yields results within 2 hours, coupled with the detection of rifampicine resistance [1]. The relatively high operating costs of the test and other limitations [2], are factors that hamper its use in resource-limited settings. Furthermore, the use of sputum-based tests is problematic in, for example, children and extrapulmonary TB cases, where appropriate quality sputum samples are difficult to obtain. Immunodiagnostic techniques could be valuable in such cases [3], [4], especially if they can be developed into rapid, point-of-care tests.

The most widely used TB immunodiagnostic tests, the interferon gamma (IFN-γ) release assays (IGRAs), have proven to be useful in the diagnosis of *Mycobacterium tuberculosis* (*M.tb*) infection especially in comparison to the tuberculin skin test (TST) [5]–[7]. These tests however, do not discriminate between latent *M.tb* infection (LTBI) and active TB disease and are therefore of limited value in high-burden settings with a high proportion of LTBI [8]. It has been shown that the detection of host markers other than IFN-γ in *M.tb*-specific antigen-stimulated whole blood cell cultures might be a useful approach for discriminating between LTBI and active TB disease [9]–[13]. Recently, we investigated the potential of 118 different *M.tb* infection phase-dependent antigens using a diluted whole blood assay, and identified antigen candidates – mostly resuscitation promoting factors (rpfs) and DosR regulon-encoded antigens with potential in the diagnosis of TB disease, as determined by IFN-γ measurement [14]. Using the Luminex platform, we here evaluated the levels of 12 host markers in culture supernatants that were stimulated with five of these promising diagnostic antigen candidates (Rv2389c, Rv0867c, Rv2032, Rv1737c, Rv0081), with the aim of identifying potentially useful diagnostic markers. We show differential cytokine production in response to *M.tb* infection phase-dependent antigens in participants with and without active TB which warrant further investigation of their diagnostic potential.

## Materials and Methods

### Ethics Statement

Ethical approval for this study was obtained from the Committee for Human Research of the University of Stellenbosch. All the study participants gave written informed consent for participation in the study.

### Study Participants

Participants enrolled into this study were recruited as part of the on-going Bill & Melinda Gates Foundation-funded Grand Challenges in Global Health (BMGF GC6-74) study (http://www.biomarkers-for-tb.net/) and have previously been described in [9], [14]. Briefly, all participants were recruited from the Ravensmead/Uitsig community, a high TB-endemic community [15] in Cape Town, South Africa, between October 2006 and April 2007. All TB patients were self-reporting, untreated cases with a first episode of TB and were acid fast bacilli (AFB)-positive on two sputum smears. Household contacts (HHCs) had been living in the same house as an adult TB case who was diagnosed not more than 2 months before recruitment of the contact. All HHCs had normal chest X-rays and AFB negative assisted sputum samples. All participants were between 18 and 60 years old, had negative HIV results (Abbot Determine™ HIV 1/2; Abbott, Wiesbaden, Germany), and gave written informed consent for participation in the study. Exclusion criteria for all participants included HIV infection, previous or current TB treatment, serious concomitant chronic conditions, steroid therapy within the past 6months and pregnancy. After collection of demographic data and completion of a clinical questionnaire, 10 ml of heparinized blood was collected from all participants and transported within 2 hours of collection to the laboratory where a 7-day whole blood assay (WBA) was performed as described in [14]. The TST, using 2 TU PPD RT23 (Statens Serum Institute, Copenhagen, Denmark), was performed on all HHCs (Mantoux method) after blood draw.

### Antigen Screening and Selection of Supernatants for Multiplex Cytokine Evaluation

A total of 118 *M.tb* infection phase-dependent antigens were evaluated as possible diagnostic candidates, based on the detection of IFN-γ responses in 7-day whole blood culture supernatants as reported previously [14]. Many *M.tb* infection phase-dependent antigens with diagnostic potential were identified. However, none of these antigens sufficed for diagnosis of TB disease with an accuracy of 100%, and such high accuracy was also not obtained when antigens were used in combinations [14]. We therefore investigated the levels of 11 alternative host markers in addition to IFN-γ, in aliquots of supernatants from whole blood cultures stimulated with the two most promising rpfs (Rv0867c and Rv2389c) and three DosR regulon-encoded antigens (Rv2032, Rv0081 and Rv1737c), alongside an unstimulated control and ESAT-6/CFP-10-stimulated cultures, using a customized Luminex multiplex assay.

All of the antigens evaluated in this study were recombinant proteins. As described previously [16], endotoxin contents in all antigens were below 50 IU/mg recombinant protein as tested by Limulus Amebocyte Lysate assay (Cambrex, East Rutherford, NJ), and all the proteins were further tested to exclude non-specific T cell stimulation and cellular toxicity in IFN-γ release assays.

### Luminex Multiplex Immunoassay

The levels of 12 host markers [epidermal growth factor, (EGF); fractalkine; interferon (IFN)-α2; IFN-γ; interleukin (IL) -4; IL-10; IL-12(p40); transforming growth factor (TGF)-α; tumour necrosis factor (TNF)-α; vascular endothelial growth factor (VEGF); interferon-inducible protein (IP)-10; RANTES] were determined in the selected supernatants using customized Milliplex kits (Merck Millipore, St. Charles, Missouri, USA), on the Bio Plex platform (Bio Plex™, Bio Rad Laboratories). This was done according to manufacturer’s instructions (Merck Millipore). Following previous optimizations, all supernatants were tested undiluted, in a blinded manner. All analyte levels in the quality control reagents of the kits were within the expected ranges. The standard curve for all analytes ranged from 3.2–10000 pg/ml. Bio-Plex Manager Software, version 4.1.1 was used for the analysis of bead median fluorescence intensity.

### Statistical Analysis

Statistical differences in analyte levels were evaluated by the Mann Whitney U test for non-parametric data analysis. Cut-off levels for differentiating between TB disease and no-TB were ascertained by receiver operating characteristics (ROC) analysis based on the highest likelihood ratio. The predictive abilities of combinations of antigen–specific host markers for TB disease and no-TB were investigated by performing best subsets general discriminant analysis (GDA), with leave-one-out cross validation as described previously [9]. A 5% significance level was used as guideline for determining significant associations. Data were analyzed using the GraphPad prism, version 5.00 for Windows (GraphPad Software, San Diego, California, USA) and Statistica software (Statsoft, Ohio, USA).

## Results

A total of 43 individuals (15 pulmonary TB cases and 28 HHCs) were included in the study. All antigens were evaluated in all of the 15 TB cases and in different HHCs (total of 15 for each antigen) from the 28 contacts that were randomly selected from the sample bank of the parent study. The mean age (±standard deviation) of all study participants was 31.5±15.9 years and 60.5% (26/43) were males. Of the 28 HHCs, 3 (10.7%) were not available for TST reading. Of the 26 with available TST results, 88.5% (23/26) were positive (10-mm cut-off), with a 20±8 mm mean (±standard deviation) induration of all 26 participants.

### Potential of Host Markers Produced by Unstimulated and ESAT-6/CFP-10-stimulated Blood Cells in Discriminating Individuals with or without TB Disease

When analyte levels detected in the unstimulated control supernatants in TB patients were compared to levels obtained in HHCs, significant differences were obtained for EGF, IFN-α2 and IL-4. After ROC analysis, however, the area under the ROC curve (AUC) was ≥0.70 only for EGF and IL-4. The sensitivities of the two analytes (EGF and IL-4) for TB disease were both <50%, but with specificities ≥96% (Table S1). The unstimulated control levels for the different host markers were subtracted from the antigen-stimulated responses for the respective study participants prior to further analysis of the data.

**Figure 1:**
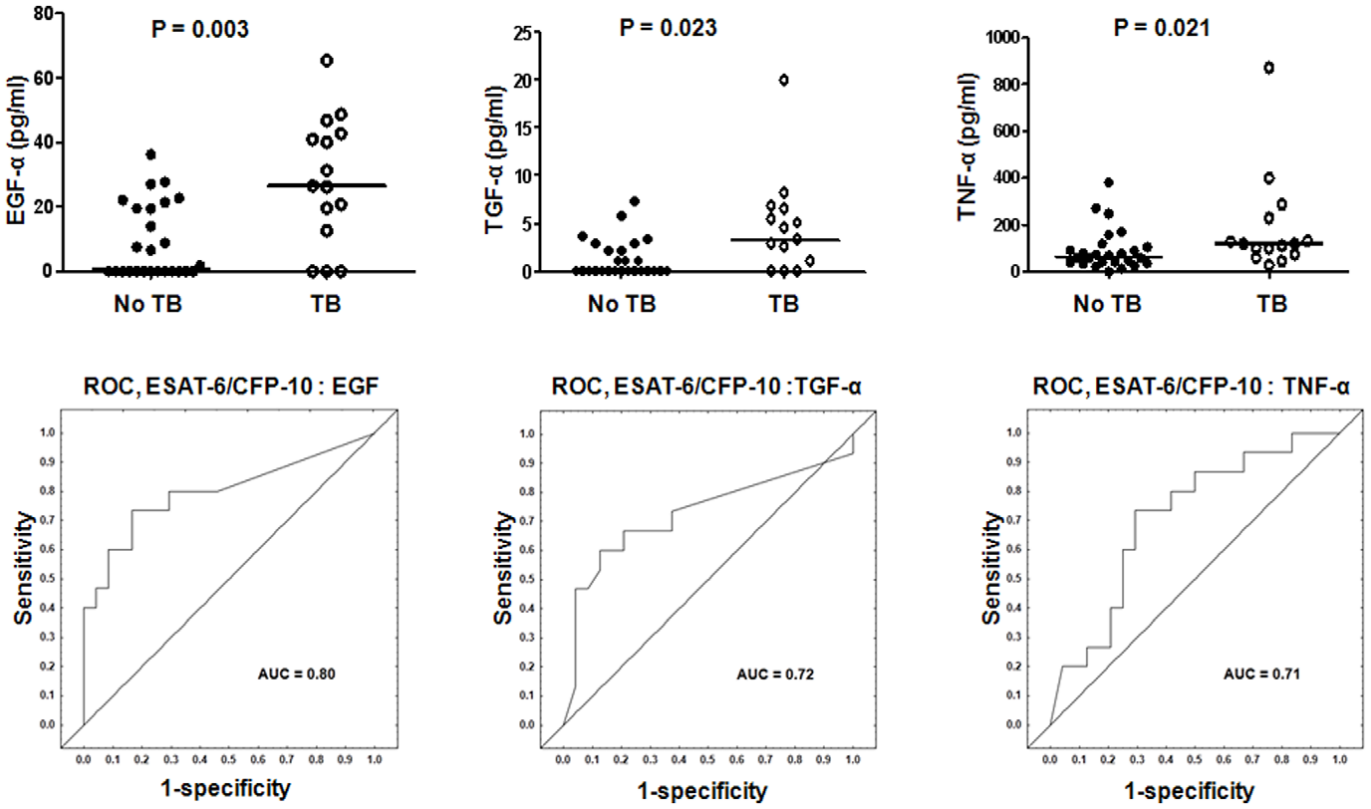
Host markers detected in ESAT-6/CFP-10-stimulated whole blood cell cultures in TB and no-TB cases and receiver operating characteristics (ROC) plots showing the accuracies of these markers in discriminating between TB disease and no-TB. Only analytes that discriminated between TB and no-TB disease with AUC ≥0.70 are shown. Error bars in the scatter dot plots represent the median analyte levels. AUC  =  Area under the ROC curve.

When ESAT-6/CFP-10 stimulated analyte levels were compared between the TB cases and HHCs, significant differences were obtained for EGF, TGF-α and TNF-α, consistent with previous observations for EGF and TGF-α in overnight whole blood assays [9]. After ROC analysis, the AUC for all of these three markers were ≥0.70. The sensitivities of the three analytes for TB disease were all <50%, but with specificities >96% (Figure 1, Table S1).

### Potential of Markers Produced by Rv0867c- and Rv2389c-stimulated Blood Cells in Discriminating Individuals with or without TB Disease

**Figure 2:**
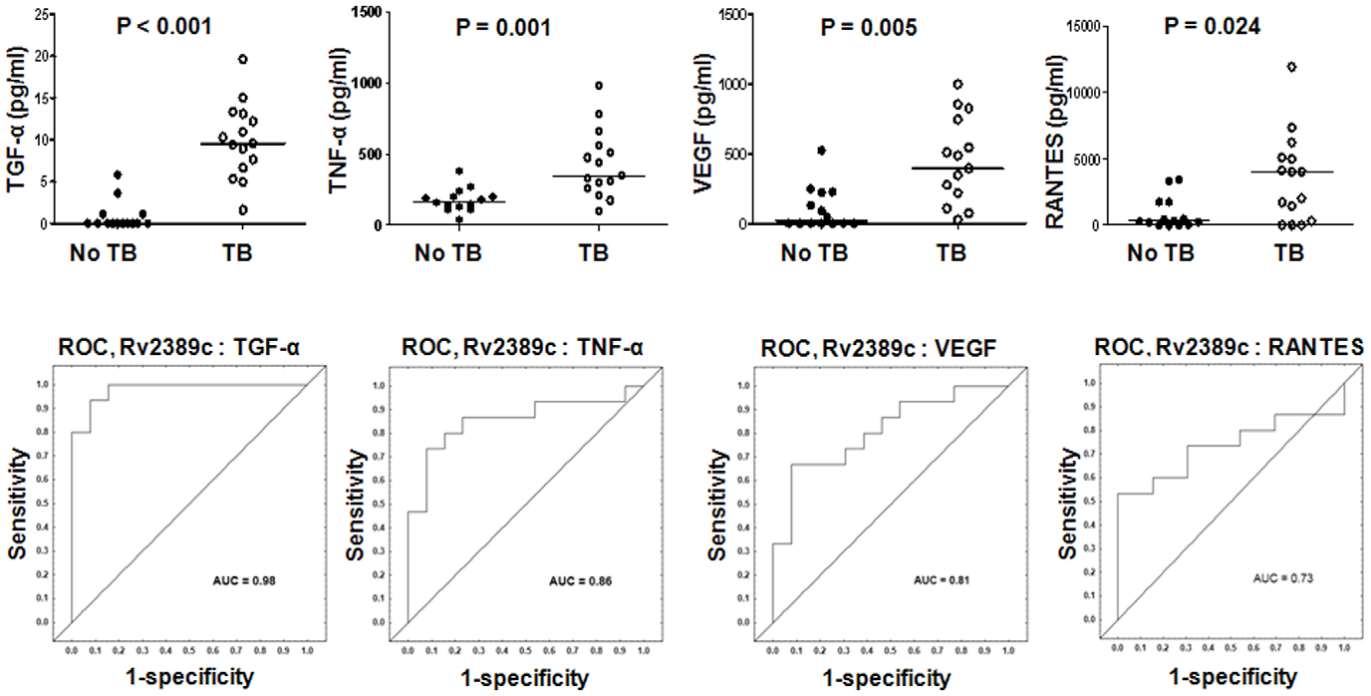
Host markers detected in Rv2389c stimulated whole blood cell cultures of TB and no-TB cases and receiver operating characteristics (ROC) plots showing the accuracies of these markers in discriminating between TB disease and no-TB. Representative plots for analytes that discriminated between TB and no-TB disease with AUC ≥0.70, are shown. Error bars in the scatter plots represent the median analyte levels. AUC  =  Area under the ROC curve.

Two of the five *M.tb* rpfs (Rv0867c and Rv2389c) were evaluated in this study. Although IFN-γ responses to the two rpfs were significantly higher in HHCs in comparison to TB cases as previously observed in the IFN-γ ELISA based study [14], the median levels of most of the other markers were higher in TB cases (Table S1). Of the other 11 host markers evaluated in Rv0867c-stimulated supernatants, TGF-α was the only additional marker (other than IFN-γ) that was significantly different between the TB cases and HHCs (Table S1). For Rv2389c-stimulated supernatants, significant differences between the TB cases and HHCs were obtained for five additional markers, namely, TGF-α, TNF-α, VEGF, IL-10 and RANTES. Unlike IFN-γ, the median levels of these markers were higher in TB cases (Table S1). After ROC analysis, the AUC for these markers (discriminating between TB disease and no-TB) was ≥0.70, with TGF-α differentiating between the two groups with both sensitivity and specificity >92% (Table S1, Figure 2).

### Potential of Host Markers Produced by Rv2032-, Rv1737c- and Rv0081-stimulated Blood Cells in Discriminating Individuals with or without TB Disease

**Figure 3:**
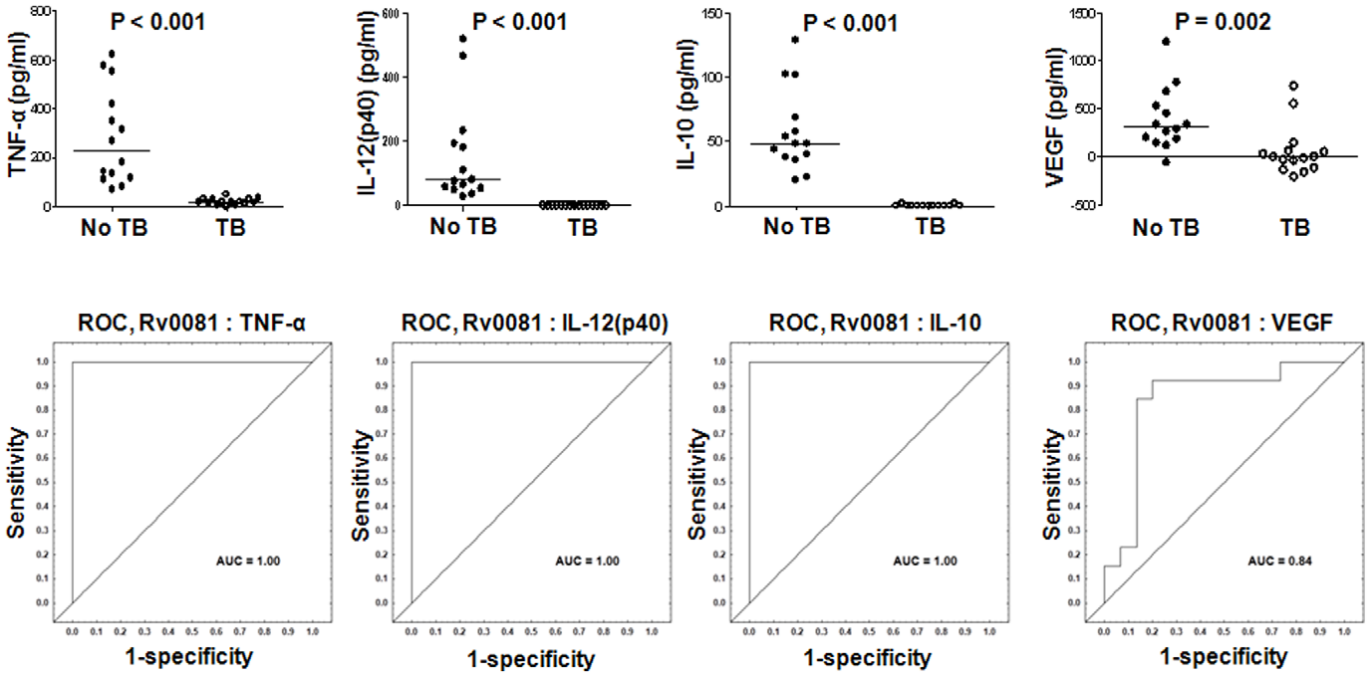
Host markers detected in Rv0081-stimulated whole blood cell cultures of TB and no-TB cases and receiver operating characteristics (ROC) plots showing the accuracies of these markers in discriminating between TB disease and no-TB. Representative plots for the most potentially useful analytes are shown. The unstimulated control levels of the markers with negative responses were higher than the analyte levels detected in Rv0081 stimulated culture supernatants. Error bars in the scatter plots represent the median analyte levels. AUC  =  Area under the ROC curve.

Three DosR-regulon-encoded antigens (Rv2032, Rv1737c and Rv0081) were evaluated in this study. The levels of eight out of the 12 markers evaluated in this study (IFN-γ, IFN-α2, IL-12(p40), IP-10, TNF-α, VEGF, IL-10 and RANTES), were significantly higher in the HHCs than the TB cases in Rv0081-stimulated supernatants (Table S1). AUC was ≥0.84 for all of these analytes, with IP-10, IL-10, TNF-α and IL-12(p40) discriminating between TB cases and HHCs with a sensitivity and specificity of 100% (Table S1, Figure 3).

**Figure 4:**
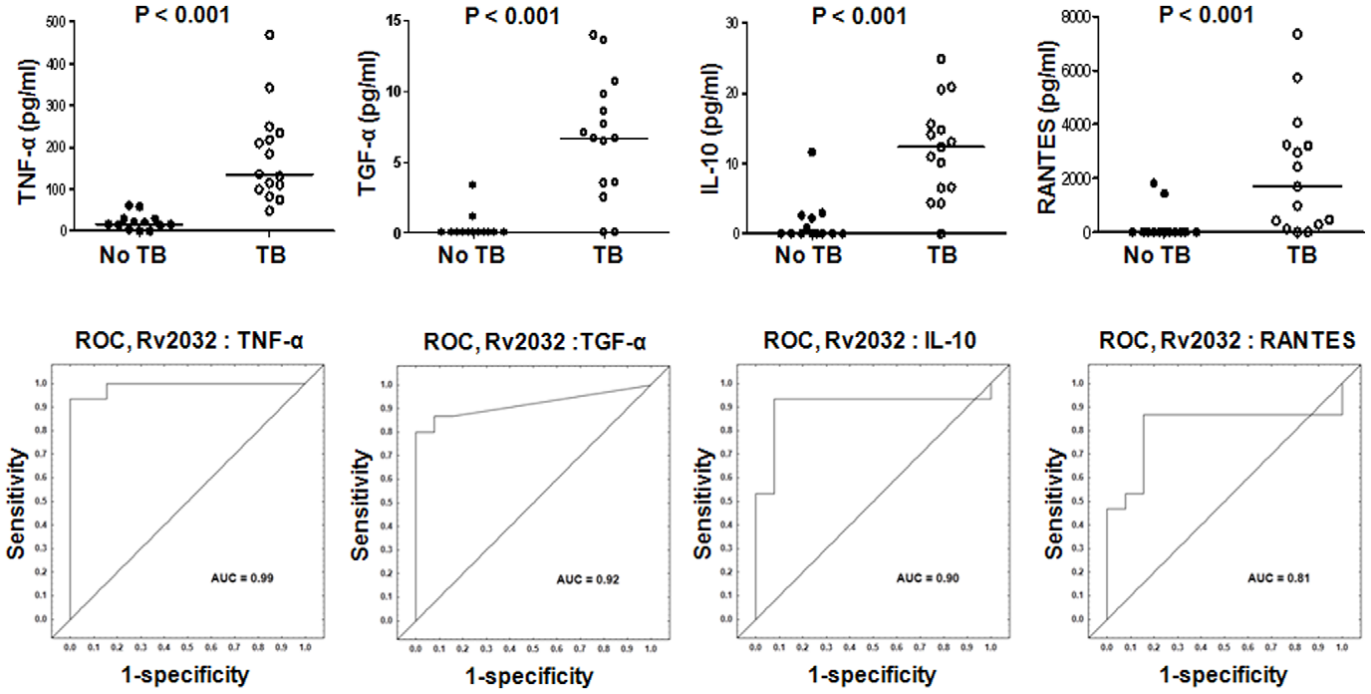
Host markers detected in Rv2032-stimulated whole blood cell cultures of TB and no-TB cases and receiver operating characteristics (ROC) plots showing the accuracies of these markers in discriminating between TB disease and no-TB. Representative plots for analytes that discriminated between TB and no-TB disease with AUC ≥0.70 are shown. The unstimulated control levels of the markers with negative responses were higher than the analyte levels detected in Rv2032 stimulated culture supernatants. Error bars in the scatter plots represent the median analyte levels. AUC  =  Area under the ROC curve.

For Rv2032, the levels of seven markers (fractalkine, IL-12(p40), TGF-α, TNF-α, VEGF, IL-10, RANTES) were significantly higher in TB cases than HHCs. These seven analytes discriminated between TB and no-TB disease with an AUC ≥0.75, and TNF-α, TGF-α and IL-10 ascertaining TB disease with both sensitivity and specificity of ≥85% (Figure 4, Table S1).

**Figure 5:**
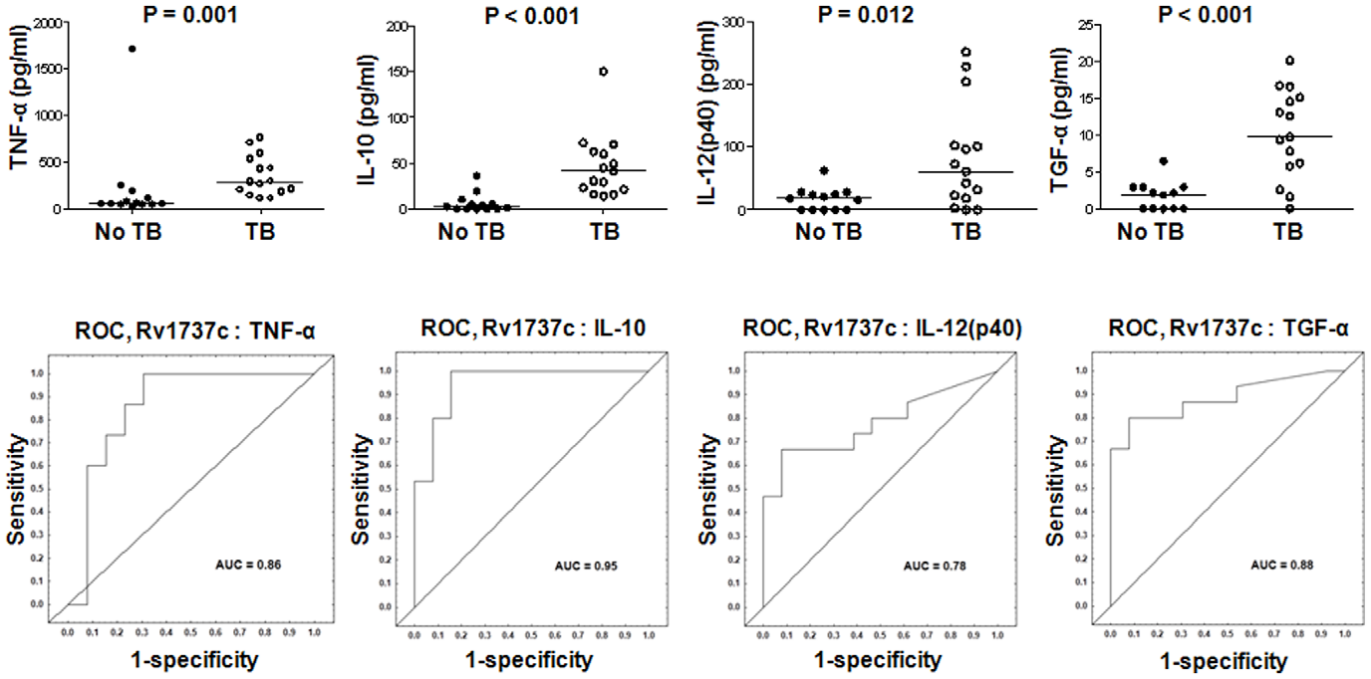
Host markers detected in Rv1737c-stimulated whole blood cell cultures and receiver operating characteristics (ROC) plots showing the accuracies of these markers in discriminating between TB disease and no-TB. Only the top 4 Rv1737c-specific analytes are shown. Error bars in the scatter dot plots represent the median analyte levels. AUC  =  Area under the ROC curve.

Similarly, Rv1737c-specific levels of IL-10, TGF-α, TNF-α, IL-12(p40) and EGF were significantly higher in the TB cases in comparison to HHCs. After ROC analysis, the AUC for all five analytes was ≥0.72 (Figure 5, Table S1).

### Utility of Analyte Combinations in Discriminating between TB and no-TB Disease

Evidence has been presented that different antigens are differentially recognised in different populations [17]. Considering that even the most promising antigens identified in that study were not recognized in all populations that were studied, and that useful diagnostic tests should perform reliably in all populations, we evaluated the performance of antigens in combination. A general discriminant analysis (GDA) [9] procedure was performed in two steps, firstly for the unstimulated and ESAT-6/CFP-10-stimulated responses, and then for all other antigen (Rv0867c, Rv2389c, Rv0081, Rv2032 and Rv1737c)-induced markers.

**Figure 6:**
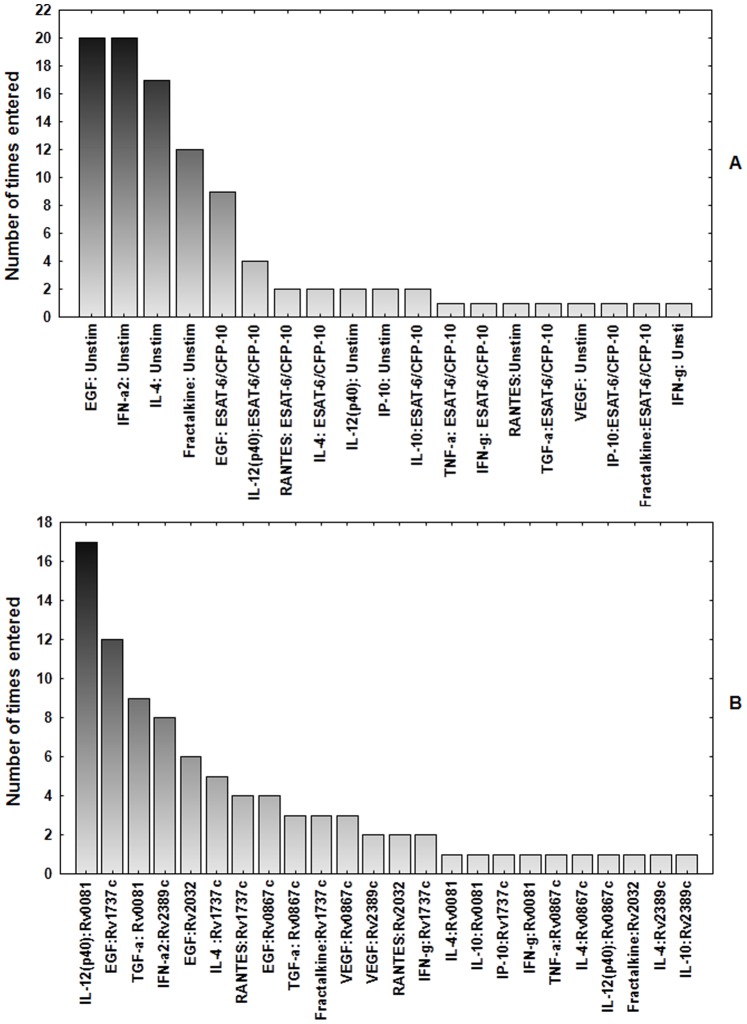
Number of times each analyte was included in the best 20 general discriminant analysis models that best classified the TB cases and HHCs into their respective groups. A, Analytes detected in the unstimulated and ESAT-6/CFP-10 stimulated supernatants. B, Analytes detected in Rv0867c-, Rv2389c-, Rv2032-, Rv0081- and Rv1737c-stimulated supernatants.

For combinations between the unstimulated control and ESAT-6/CFP-10-specific analytes, different five-antigen-host marker combination models resulted in the accurate prediction of ≥80% of the TB cases and ≥90% of the HHCs after leave-one-out cross validation (data not shown). The top antigen–host marker combination (unstimulated EGF + unstimulated fractalkine + unstimulated IFN-α2+ unstimulated IL-4+ ESAT-6/CFP-10-specific RANTES) accurately predicted 87% of TB cases and 100% of HHCs. The host markers that were included most frequently among the top 20 GDA -marker combinations were unstimulated EGF and unstimulated IFN-α2 (Figure 6). For the combinations between Rv0867c, Rv2389c, Rv0081, Rv2032 and Rv1737c-specific analyte levels, all the top 20 five-antigen-specific host marker combinations accurately predicted all (100%) of the TB cases and HHCs after leave-one-out cross validation (data not shown). The most predictive analytes in the top 20 analyte combination models included Rv0081-specific IL-12(p40), Rv1737c-specific EGF and Rv0081-specific TGF-α levels (Figure 6).

## Discussion

IFN-γ remains the primary marker in T-cell stimulation assays, due to its critical role in the development of *M.tb*-specific acquired immune responses [18]. In this study, we show that stimulation of whole blood cells from TB patients and HHCs with different *M.tb* infection phase-dependent antigens, results in the production of multiple host markers other than IFN-γ. In addition to confirming results obtained with IFN-γ in previous studies, we show that several, non-canonical analytes are promising markers of active TB disease. *M.tb* DosR regulon-encoded Rv0081 antigen-specific induced levels of IL-12(p40), IP-10, IL-10 and TNF-α were the most promising diagnostic candidates in this study. Combining different *M.tb* antigens and host markers may improve diagnostic ability substantially.

Much progress has been made in the development of new and more effective diagnostic tools for TB, notably IGRAs and the more recently developed XpertMTB/RIF assay. In spite of this progress, the diagnosis of TB disease in resource-constrained settings, which often bear the highest TB and HIV co-infection burden, especially Sub-Saharan Africa [19], [20], remains challenging. This is mainly because of the continued reliance in these settings on smear microscopy and empirical clinical diagnosis due to the lack of proper laboratory diagnostic tools [19]. Such settings would benefit from a rapid and robust field-friendly point-of-care test, especially if such a test proves to be highly accurate in the diagnosis of active TB disease in areas with concurrently high LTBI rates. The host markers evaluated in this study are pro-inflammatory and anti-inflammatory markers that have been widely investigated for different purposes in different infectious diseases, including TB. The uniqueness of our study, however, is the fact that these markers were evaluated in supernatants of whole blood cultures that were stimulated with recently identified *M.tb* infection phase-dependent antigens, and that these markers are of potential value for the diagnosis of TB disease in a highly endemic setting. This study was conducted in an area where the prevalence of LTBI is very high, as evidenced by high IGRA positivity in previous studies [6], [7], [9], [17], and the >88% TST positivity of the non TB cases in this study. Thus, these novel *M.tb* antigen-specific markers may be useful in discriminating between LTBI and active TB disease.

The concept that stimulation of whole blood cells from *M.tb*-infected or -diseased individuals with *M.tb* antigens results in the secretion of multiple host markers, that could be useful diagnostic markers is not new as such markers have been investigated in other studies [9], [12], [13], [21]–[29], including studies on bovine TB [30]. These mostly ESAT-6/CFP-10± TB7.7 -based studies have implicated some of the markers investigated in this study, notably TNF-α and IP-10 (*reviewed in* [31]) [22], [25]–[27], [29] as adjunct markers for use singly or in combination with IFN-γ in the diagnosis of *M.tb* infection; or as potential candidates for discriminating between LTBI and active TB disease (IP-10, TNF-α, IL-12(p40), EGF, TGF-α, and VEGF) [9]–[13]. The results obtained for the ESAT-6/CFP-10-specific levels of EGF, TGF-α, and the unstimulated EGF responses in this study are in agreement with previous observations in the same study population [9]. Stimulation of whole blood with other antigens such as TB10.4, coupled with the detection of host markers such as TNF-α, IL-17, and IL-12(p40) has also been shown to be potentially useful in the diagnosis of TB disease in a West African setting [13]. Out of 29 host markers, including most of the markers evaluated in this study, that were investigated for discrimination between LTBI and TB disease in children in a low burden setting however, none of the markers, with the exception of IL-2, was significantly different between latently infected children and those with TB disease [11]. It is not known whether this difference in the studies could only be explained by factors such as age of study participants (children versus adults), geographical location, bacterial strain differences and other factors including genetic differences in HLA genes [32]. It is worth mentioning however, that data on host markers for discriminating between TB disease and LTBI are currently very limited, extremely heterogeneous, and such studies have so far been performed only in limited participant numbers, partly because of the cost of multiplex immunoassay kits as acknowledged in [9], [22]. Furthermore, the potential for false discoveries also exists, given the large numbers of markers that are often evaluated and the small patient numbers in these studies. More data on the most promising markers that have been described to date are needed before their diagnostic potential can be verified. However, suitable selection criteria for future qualification of promising markers are currently not defined but will be absolutely necessary to allow affordable evaluation of candidate markers.

Little is known about the relatively new *M.tb* infection phase-dependent antigens investigated in this study (Rv0081, Rv2032, Rv0867c, Rv2389c, Rv1737c), notably about their immunogenicity in LTBI or TB disease. In the few studies that have investigated these antigens however, it has been shown that IFN-γ responses to both the rpfs and DosR regulon-encoded antigens are strongly associated with latency [33]–[35]. Furthermore, the rpfs and DosR regulon-encoded antigens have also been shown to elicit marked CD4 and CD8 T cell responses in vitro, in long-term latently infected individuals [35], [36]. Our recent report [14] based on IFN-γ measurement in culture supernatants by ELISA implicated Rv0081, Rv2032, Rv0867c, and Rv2389c as promising diagnostic antigen candidates. In spite of the promise shown by these antigens as possible TB diagnostic candidates none of the antigens discriminates between TB and absence of TB disease with both sensitivity and specificity >85% in IFN-γ based tests and this degree of accuracy is not achieved even when antigens are evaluated in combinations of four [14]. We have, however, shown for the first time that unlike IFN-γ for which higher levels are observed in latently infected individuals upon stimulation of whole blood with these antigens, the levels of most other markers were higher in patients with active TB disease with the exception of Rv0081-specific markers, which were generally higher in the HHCs. Work done with other DosR regulon-encoded antigens, such as Rv2628, although not investigated in this study, has shown that responses against this antigen are associated with cured TB and remote latent infection [37], and higher frequencies of T cell responses to this antigen occur at the site of disease [38]. Our data indicate that measurement of host markers such as IL-12(p40), IP-10, IL-10 and TNF-α upon stimulation with even a single antigen, such as Rv0081, could be sufficient in diagnosing TB disease with high accuracy in a setting with high LTBI.

Of all the 12 markers evaluated in this study, the levels of 11 (IL-12(p40), IFN-γ, IP-10, TNF-α, IL-10, TGF-α, IFN-α2, RANTES, VEGF, fractalkine, EGF) after background correction, were significantly different between the TB and no TB cases following stimulation with at least one of the six antigens investigated. IL-12(p40), TNF-α, IL-10 and IP-10, a chemokine produced by many cell types including monocytes, endothelial cells, neutrophils amongst others in response to IFN-γ [31], were amongst the best candidate diagnostic markers identified in this study. The median levels of these markers in Rv0081 stimulated supernatants, just like the levels of IFN-γ, were higher in the non TB cases. From what is known about the biological roles of these markers (all Th1 immune response and/or granuloma formation favourable signals; with the exception of IL-10 which is anti-inflammatory) [39], [40], it is not surprising that the induced levels of the markers in Rv0081 stimulated supernatants all correlated significantly with IFN-γ (r, 0.40–0.62; p, 0.02–<0.001). Although the levels of these markers were higher in the non TB cases in Rv0081 stimulated supernatants, higher levels of the markers were observed in the TB cases upon stimulation with the other DosR regulon-encoded antigens (Rv2032 and Rv1733c), and correlations between the four markers (IL-12p40, IL-10, IP-10, TNF-α) and IFN-γ were only significant for IL-12(p40) (r, 0.43; p, 0.02) in Rv2032 stimulated supernatants, and IP-10 (r, 0.5; p, <0.01) in Rv1737c stimulated supernatants. The generally higher Rv2032- and Rv1733c-specific marker responses in TB cases were more similar to what was observed in the rpf (Rv2389c and Rv0867c) -stimulated supernatants. The meaning of this differential marker induction pattern, even in antigens that are expressed in the same phase of *M.tb* infection, is not clear as data on these new antigens is still scanty. IFN-γ responses against all the antigens however, were as observed in the recent IFN-γ ELISA based study on the same participant cohort [14], and in agreement with previous observations [33]–[35]. Future validation studies will determine which of the antigen–specific markers analyzed here possesses the best diagnostic potential, given that the diagnostic potential of these *M.tb* antigens and host markers can be maintained in overnight assays. These antigen–specific host markers may provide the basis for the development of a novel TB diagnostic test, highly suitable for high TB burden areas.

The molecular beacon assays such as the GeneXpert test provide results within 2 hours. Yet, the strength of an immunodiagnostic modality is that such an assay could be more practical in high TB burden and resource-limited settings, considering the obstacles involved in the use of the GeneXpert and similar tests that rely on advanced laboratory equipment as highlighted by Trebucq et al [2]. If the diagnostic potential of the antigen specific host markers investigated in this preliminary study is maintained in larger validation studies, T cell based immunodiagnostic modalities might be developed, that involve the coating of blood collection tubes with the antigens for short-term culture, as is currently done with the Quantiferon TB Gold In Tube system. Antibodies against the target host markers could then be incorporated into a dip-stick-like test membrane which would then be inserted into the supernatant after culture and results read in 10 to 20 minutes. Such lateral flow test devices are currently being evaluated for the diagnosis of many infectious diseases including TB [41]–[44], and these assays have the potential to detect multiple host markers, including some of the markers evaluated in this study, in culture supernatants [43]. Although such a diagnostic modality will only yield results within 24 hours after the patient’s visit, it might prove cheaper and more suitable in remote settings than the GeneXpert and conventional *M.tb* culture which has been the gold standard for a long time, but yet unavailable in these resource-limited settings. Such a test would also be beneficial to individuals that have difficulty in providing satisfactory sputum samples for microbiological and other tests such as children and individuals with extrapulmonary TB.

One concern relating to the development of lateral flow based tests is the ability to detect markers that are produced in low amounts. It has been shown that cytokine responses can be enhanced by addition of cytokines such as IL-7 [45] and IL-12 [46] into cultures and through the delivery of the antigens using vectors such as the *Bordetella pertusis* adenylate cyclase vector [47], and other adjuvants [48]. Lateral flow tests using upconverting phospor technology were shown to detect IFN-γ in culture supernatants with sensitivity below 2 pg/ml and were more sensitive than ELISA in detecting *Shistosoma* circulating antigens [44]. Such considerations will only become relevant if diagnostic ability of such markers is validated in appropriate larger studies.

As previously reported [14], a serious concern in the possible use of new antigens such as those investigated in this study for diagnostic purposes is a lack of specificity for *M.tb*. Some DosR regulon-encoded antigens have been shown to be expressed in BCG [49], which could potentially affect the value for diagnosis in BCG-vaccinated populations. However, BCG vaccination was found not to induce T cell responses to DosR regulon-encoded antigens, likely because BCG fails to establish long-lasting latent infection and therefore may not express these antigens in vaccinated individuals [50]. Future studies also need to evaluate specificity in lung inflammation other than TB as levels of several cytokines in unstimulated and/or ESAT-6 stimulated whole blood samples have been shown to not be useful in discriminating between TB disease and pneumonia [23].

### Study Limitations

The main limitations of our study include the small sample size and the use of a 7-day whole blood assay instead of an overnight assay which would be more suitable for a diagnostic test. Another limitation of our study is the potential of reporting a significant finding which occurred by chance, given that 12 host markers were evaluated in 5 different *M.tb* infection phase-dependent antigen-stimulated supernatants. Such a risk applies in all biomarker discovery studies regardless of the discovery platform used. One corrective measure that is usually employed during statistical analysis is correcting for multiple comparisons. The main analytical procedure employed in this study was ROC analysis, a method in which no hypothesis testing is done, the decisions rather resulting from likelihood ratios [51]. Not correcting for multiple comparisons in this study however, may be a concern in GDA as the best subsets method (applied in this study) does generate different analyte combinations. However, as we did not compare different combinations of analytes and rather evaluated the frequency of inclusion of individual markers into the top 20 models that were generated by GDA (Figure 6), correction for multiple comparisons does not apply.

Future evaluation of the antigens and host markers identified in this study should be done in larger numbers of participants, in overnight assays to detect effector cell responses, and preferably using a prospective study design. Future studies should also evaluate the antigens and host markers in children, immunocompromised individuals, extrapulmonary TB cases, and also in individuals with other lung diseases [23]. A diagnostic modality derived from these antigens and host markers would be highly valuable for TB control if the host markers could be incorporated into a rapid, lateral flow test device.

### Conclusions

In conclusion, the measurement of host markers (other than IFN-γ) in supernatants after stimulation with *M.tb* infection phase-dependent antigens promises to be a useful method of diagnosing TB disease. Rv0081-specific levels of IP-10, IL-12(p40), IL-10 and TNF-α, Rv2389c-specific levels of TGF-α, and Rv2032-specific levels of TNF-α showed the most promise as TB diagnostic candidates. The results of this study are preliminary and warrant further investigation in larger studies and in other settings.

## Supporting Information

Table S1 Median levels of all analytes evaluated (pg/ml) and ranges (in parentheses) elicited upon stimulation of whole blood cells with different *M. tuberculosis* infection phase-dependent antigens and abilities to discriminate between pulmonary TB disease (in 15 cases) and no TB (in 15 household contacts). AUC, area under the curve; 95% CI = 95% confidence interval. The p-values shown are Mann Whitney U test P values for differences between TB cases and HHCs. The cut-off values are for the sensitivity and specificity for TB disease. Antigens and host markers were sorted according to AUC (highest to lowest) for discriminating between TB and no-TB disease.
(XLS)
