# Correction: Potential of Host Markers Produced by Infection Phase-Dependent Antigen-Stimulated Cells for the Diagnosis of Tuberculosis in a Highly Endemic Area

**DOI:** 10.1371/annotation/bc36a9c6-d5c0-4d55-bc92-9ce4a07b4f70

**Published:** 2012-08-31

**Authors:** Novel N. Chegou, Paulin N. Essone, Andre G. Loxton, Kim Stanley, Gillian F. Black, Gian D. van der Spuy, Paul D. van Helden, Kees L. Franken, Shreemanta K. Parida, Michel R. Klein, Stefan H. E. Kaufmann, Tom H. M. Ottenhoff, Gerhard Walzl

There were errors in the Funding section. The correct funding information is as follows: This work was supported by the Bill & Melinda Gates Foundation through Grand Challenge 6 (BMGFGC6, grant no 37772); and the EDCTP through the African European Tuberculosis Consortium (AE-TBC, grant number IP_2009_32040) and the Trials of Excellence in Southern Africa (TESA, project code CG_cb_07_41700). Dr Chegou received financial support from the Claude Leon Foundation and the South African MRC during the writing up of this work. The funders had no role in study design, data collection and analysis, decision to publish, or preparation of the manuscript. 

